# A Low-Power Wireless System for Predicting Early Signs of Sudden Cardiac Arrest Incorporating an Optimized CNN Model Implemented on NVIDIA Jetson

**DOI:** 10.3390/s23042270

**Published:** 2023-02-17

**Authors:** Venkata Deepa Kota, Himanshu Sharma, Mark V. Albert, Ifana Mahbub, Gayatri Mehta, Kamesh Namuduri

**Affiliations:** 1 Department of Electrical Engineering, University of North Texas, Denton, TX 76203, USA; 2 Department of Computer Science and Engineering, University of North Texas, Denton, TX 76203, USA; 3 Department of Electrical and Computer Engineering, The University of Texas at Dallas, Richardson, TX 75080, USA

**Keywords:** ECG, wearable, wireless, low-power, transceiver, CNN

## Abstract

The survival rate for sudden cardiac arrest (SCA) is low, and patients with long-term risks of SCA are not adequately alerted. Understanding SCA’s characteristics will be key to developing preventive strategies. Many lives could be saved if SCA’s early onset could be detected or predicted. Monitoring heart signals continuously is essential for diagnosing sporadic cardiac dysfunction. An electrocardiogram (ECG) can be used to continuously monitor heart function without having to go to the hospital. A zeolite-based dry electrode can provide safe on-skin ECG acquisition while the subject is out-of-hospital and facilitate long-term monitoring. To the ECG signal, a low-power 1 μW read-out circuit was designed and implemented in our prior work. However, having long-term ECG monitoring outside the hospital, i.e., high battery life, and low power consumption while transmission and reception of ECG signal are crucial. This paper proposes a prototype with a 10-bit resolution ADC and nRF24L01 transceivers placed 5 m apart. The system uses the 2.4 GHz worldwide ISM frequency band with GFSK modulation to wirelessly transmit digitized ECG bits at 250 kbps data rate to a physician’s computer (or similar) for continuous monitoring of ECG signals; the power consumption is only 11.2 mW and 4.62 mW during transmission and reception, respectively, with a low bit error rate of ≤0.1%. Additionally, a subject-wise cross-validated, three-fold, optimized convolutional neural network (CNN) model using the Physionet-SCA dataset was implemented on NVIDIA Jetson to identify the irregular heartbeats yielding an accuracy of 89% with a run time of 5.31 s. Normal beat classification has an F1 score of 0.94 and a ROC score of 0.886. Thus, this paper integrates the ECG acquisition and processing unit with low-power wireless transmission and CNN model to detect irregular heartbeats.

## 1. Introduction

In the United States, 200–450 thousand adults die yearly from sudden cardiac death (SCD). Annually, approximately 350,000 are out-of-hospital cardiac arrests (OHCA), according to the American Heart Association’s 2020 report [1]. Statistics based on research reveal only a 10% chance for the out-of-hospital SCA survival rates [2]. Sixty-five percent of these OHCAs occur at home or in residence [3]. For accurate prediction of sudden cardiac arrests (SCAs), it is imperative to recognize high-risk patients and provide immediate treatment. Due to the retrospective nature of sudden cardiac arrest categorization, it is difficult to determine the circumstances surrounding the death [4]. Research suggests that there are many instances where discrepancies occur but are not diagnosed, resulting in SCD [5]. Several ECG markers have been identified as potential SCA markers, including *QT* prolongation, *QRS* duration, fragmented *QRS* complexes, early re-polarization, signal-averaged ECGs, *T*-wave alternans, and *T*-peak to *T*-end that may occur repetitively [6]. The timely intervention has an increased chance of survival, and it is possible to predict SCA in the early stages of Ventricular Fibrillation (VF) through continuous monitoring [7]. There is a dire need for an effective and efficient life-saving system to detect and prevent SCA. A functional wireless ECG system can facilitate monitoring of the heart’s activity outside the clinical setting. It also allows for continuous (≥24 h) monitoring of heart function, thus enabling detection and diagnosis of SCA at its early onset. One of the main challenges for the early detection and prevention of SCA is developing a ubiquitous, continuous, and efficient ECG transmission that is secure and reliable. The requirement is a wireless ECG monitoring system that provides an unlimited monitoring period, decreases the time to diagnosis, and improves patient care significantly.

There has been prior work on wireless ECG transmission using Arduino boards and RF wireless modules. Güvenç et. al. provides a detailed transmitter and receiver design, notes, and connections between Arduino Mega 2560 boards (Manufacturer: Arduino) and nRF24L01, (Manufacturer: Nordic Semiconductor ASA), focusing on minimization of the noise factor during wireless transmission [8]. However, performance parameters such as power consumption, data rate, the distance between transmitter and receiver, and bit error rate are not measured [9]. For continuous ECG monitoring, battery life and signal integrity are critical. Previous research has developed a wireless transmission through Bluetooth technology. This work uses the MAX30003 (Manufacturer: Analog Devices) system on a chip, which has an amplifier, filters, and processing components best suited for analog time-series signals such as ECG. MAX30003 is a large, bulky unit measuring 3.5 × 1.5 inches, and Bluetooth technology increases power consumption due to a current of 600 mA [10]. A miniaturized, portable wireless transmission with at least a week (≥24 h) of uninterrupted battery life is a factor to consider. Kumar et. al. has provided the current during wireless transmission and reception with a data rate of 1–2 Megabits per second (Mbps). However, the transmission range and the signal transmission accuracy are not measured [11]. An efficient ECG wireless system requires the transmission distance and the effect on the bit error rate. We aim to build a wireless system with low-power consumption and test continuous ECG transmission and reception over a small distance within lab premises with a negligible bit error rate to detect early signs of SCA. In addition to transmission, it is required to process the continuous ECG data collected over time. A machine learning (ML) model is well-suited and can easily handle extensive time-series-based biosignals such as ECG. The software implementation of the ML model, which includes training and testing on the ECG data with high accuracy, will benefit Physicians in processing data. Until now, there has been no test or system to predict when SCA will occur and who is at high risk for needing a doctor’s attention. A portable hardware unit must implement the software ML model to quickly process the large ECG data in real-time and may be combined with a wireless SCA prediction system. The choice of the ML model is an important factor for an efficient system. Prior research indicates that simulation-based ML helps predict out-of-hospital cardiac arrests. Murugappan et. al. proposed a classification model using MIT-BIH SCA data. These data contain four non-linear features: approximate entropy (ApEN), Hurst exponent, sample entropy (SampEN), and the most prominent Lyapunov exponent (LLE) are calculated from 1-min ECG segments to predict SCA occurrence before the VF onset (actual SCA trigger point). This work recommends a support vector machine (SVM) as the best-suited model with a maximum mean classification rate of 100%. A comparison between the SVM model and a neural network concludes that a neural network increases the complexity and is not the best option for ECG heartbeat classification [5]. However, the non-linear feature calculation at the receiver end requires additional processing and is a time-consuming part, delaying the inputs to the ML model. Shen et. al. utilized four-time domain features with a Multi-Layer Perceptron (MLP) algorithm and reported 67.44% accuracy in predicting SCA using heart rate variability (HRV). The model’s accuracy is low and did not discuss the reasons for choosing the MLP model. Hardware implementation of the MLP model is not discussed. Another HRV-based prediction model was developed for detecting SCA at an early stage with an accuracy of 86.8% and 94.7% using the k-Nearest Neighbor (kNN), and support vector machine (SVM) classifiers [12]. The disadvantage of HRV signals is that their processing is computationally intensive [7]. Also, the detection of SCA is not accurate and dependent on HRV data. ECG abnormality detection has recently been based on neural network algorithms. Prior work with an improved prediction performance used a CNN model on data collected from the Pediatric ICU at the Hospital for Sick Children in Toronto. By aggregating risk predictions, cardiac arrest prediction was made possible. However, the model has limited applications and cannot be applied to new subjects [13]. Our purpose is to build an optimized convolutional neural network (CNN) model that will enhance SCA prediction from the ECG data of any new person.

The primary contribution of this paper is to build a prototype for wireless transmission of digitized ECG bits using a microcontroller and transceiver and test the prototype within the lab for efficient communication and reception. The wireless module combines our prior work on a readout circuit and zeolite-based dry electrodes for a complete ECG wireless SCA prediction system. The first work to demonstrate and test a wireless transmission of ECG bits at a 250 kbps data rate, determine the power consumption along with bit error rate percentages (loss of bits or improper communication), and demonstrate SCA heartbeat classification on a portable NVIDIA Jetson Nano System-on-Module. Thus, the paper’s novelty is integrating an ECG wireless transmission system with a CNN model toward building a complete SCA heartbeat classification system best suited for a new subject’s ECG data.

**Figure 1:**
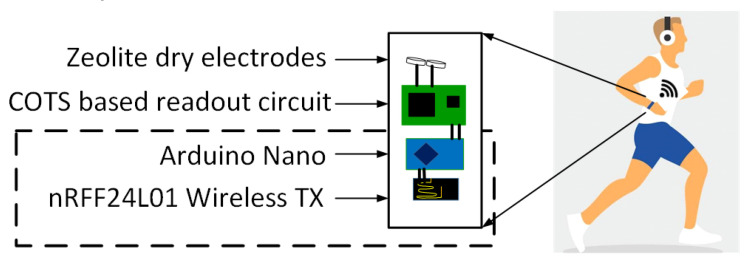
Overview of the ECG wireless SCA transmission system.

The overview of the compact wireless ECG transmission system is shown in Figure 1, in which a wireless module, using the analog-digital converter (ADC) unit from the Arduino Nano microcontroller and RF Transceiver (nRF24L01), is shown using a dotted line. The transceiver part is the current focus of this paper. Our prior work involving the FR4 substrate-based readout circuit and zeolites-based dry electrodes is used to acquire and pre-process the ECG data [14,15]. In recent research, it was found that a smartwatch worn on the hand was capable of detecting abnormalities in the heart. This was based on a statistical analysis of healthy subjects and patients [16]. The paper is organized as follows: Section 2 discusses the wireless system design, experimental setup, SCA dataset, and the machine learning model; Section 3 discusses the results of the wireless system; and the optimized CNN model with classification accuracy, F1 score; lastly Section 4 presents the concluding remarks.

## 2. Design and Experimental Setup

### 2.1. Wireless ECG Module

**Figure 2:**
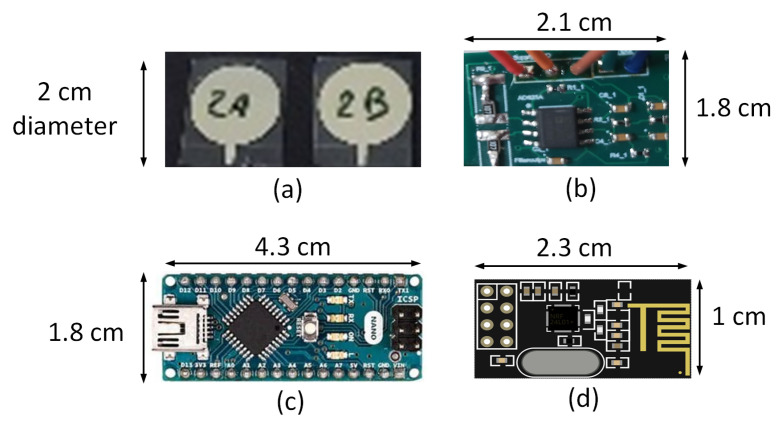
(**a**) Zeolite-based dry electrodes (**b**) Read-out-circuit (**c**) ADC (**d**) nRF24L01 wireless transreceiver.

**Figure 3:**
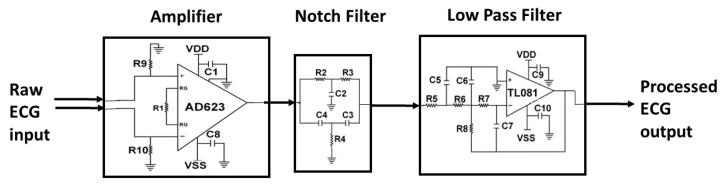
The core PCB circuit diagram.

**Figure 4:**
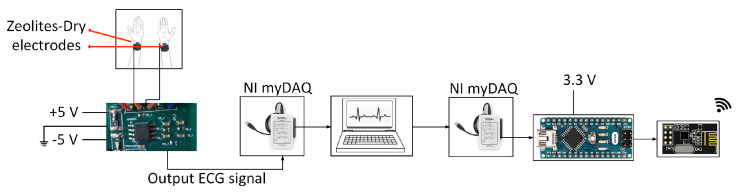
Experimental setup for ECG wireless transmission.

SCA is characterized by abnormal ECG signals that occur without prior indication, often when patients are outside hospitals. Ideally, low-power (long battery life), low-cost, and miniaturized compact ECG recording systems enable chronic disease management and preventive healthcare with a bit error rate of <10% [17]. An ECG monitoring system ideally begins with collecting ECG data from a human subject. Zeolite and polydimethylsiloxane (PDMS) composite dry electrodes are used for acquiring ECG signals. The primary advantage of the zeolite-based dry electrode is user comfort and reliable long-term ECG acquisition. Dry electrodes using 13X Zeolite-PDMS with a diameter of 2 cm and a filler concentration of 12% *w*/*w* are proposed and are illustrated in Figure 2a. Placing the electrodes in the precordial position, ECG was acquired from a subject while strolling (limited to 20 steps) [15]. The weak ECG signal needs to be amplified and filtered for noise components before transmitting the signal. In our prior work, we designed and built a compact ECG processing unit on an FR4 substrate with three main components. An AD623 amplifier with 60 dB gain, an RC-based notch filter to remove power line interference (60 Hz), and a third-order low-pass filter using Texas Instruments’s TL081 operational amplifier to dismiss higher-order frequencies (>100 Hz) is implemented on an FR4 substrate for processing the ECG signal and is shown in Figure 2b. The specific values of the resistances and capacitances can be found in our prior work [14]. The target of the wireless ECG system is to transmit the ECG data with low power consumption ≤28 mW and low bit error rate ≤10%. This work of a wireless ECG module extends our prior ECG acquisition and ECG processing system [14,15]. For transmission, the ECG signal is digitized, and an ADC of 1.8 × 4.3 cm^2^ size from an Arduino nano microcontroller with a resolution of 10 bits, a maximum sampling rate of 1M samples per second is incorporated, as shown in Figure 2c. The advantage of Arduino is that it is low-cost, compact, and has an option to choose from 10–16 bits of resolution. For a transmitter, a low-power nRF24L01 transceiver module, of size 2.3 × 1 cm^2^, as shown in Figure 2d, is used. The core printed circuit board (PCB) circuit diagram is shown in Figure 3. The transceiver transmits data using Gaussian frequency-shift keying (GFSK) modulation. The Gaussian type of FSK modulation uses a Gaussian filter to shape the pulses before they are modulated. It is well suited for narrow bandwidth biosignals such as ECG data transmission. The transceiver operates at 2.4 GHz, with a data rate of 250 kbps. The pin configuration for nRF24L01 and connections to an Arduino are shown in Table 1. For our design, the transmitter output power is programmed to −18 dBm. The distance between the transmitter and receiver is 5 m. An nRF24L01+ can transmit up to 100 m in an open field at 250 kbps [8]. The nRF24L01 transceiver module communicates over a 4-pin serial peripheral interface (SPI) with a data rate of 250 kbps. National Instruments’s myDAQ ((Model number: 78132901) acts as a transducer to input the acquired ECG signal into the ADC of the Arduino microcontroller. The selection of input ports, and after the digitization, the flow of bits is directed to the output ports in MATLAB. Transmitting at 2.4 GHz is achieved using a single-chip radio transceiver, nRF24L01, which is compatible with Arduino. The transceiver has an option of a higher data rate (250 kbps to 2 Mbps) and transmission distance and hence is a better choice for transmitting large volumes of ECG data. The experimental setup for wireless transmission and reception of ECG bits is shown in Figure 4. The role of myDAQ is to apply the analog ECG signal to nRF24L01. A single-direction communication system is implemented in this study. The MATLAB code for transmission and reception is developed and loaded into the microcontroller using the Arduino Integrated Development Environment (IDE) interface. The module’s operating voltage is 3.3 V. However, since SCA does not have any symptoms, or early signs, the mere transmission of ECG signals is insufficient; learning SCA features is useful.

### 2.2. Physionet SCA Data

PhysioNet’s SCA database supports research and provides an opportunity to progress in the prediction of SCA. The MIT-BIH Malignant Ventricular Arrhythmia Database (collected initially by Scott Greenwald at MIT) was used to generate the SCA dataset. SCA includes half-hour excerpts from 23 Holter recordings (13 males, 8 females, and two unknown genders; ages 17 to 82). The database consists of subjects with underlying conditions: 18 patients with sinus rhythm (4 with intermittent pacing), 1 with a pacemaker, and 4 with atrial fibrillation. Each patient had a sustained ventricular tachyarrhythmia, and most suffered from a prior cardiac arrest. Twelve subjects’ ECG signals were sampled at 250 Hz and annotated by expert physicians. For each of the twelve annotated subjects, a VF onset elapsed time parameter (hh:mm:s) is listed by the Physicians suggesting the actual occurrence of a cardiac arrest [18]. There is a significant difference in the ECG signal after the VF onset compared to before the VF onset. Based on the sampling rate of 250 Hz, the heartbeats are separated, and each heartbeat is annotated. The annotations are divided into two categories: beat annotations and non-beat annotations. The annotations are labeled with an alphabet. Symbols specify the non-beat annotations, e.g., a vertical line | indicates an Isolated QRS-like artifact. The heartbeat and non-beat annotations and their description are listed in Table 2. More details about the patient’s age, prior heart condition, and heartbeat annotation have been presented in Goldberger et al. For the identification of normal and abnormal heartbeats, an expert Physician is needed. The process involving a senior Physician to separate the heartbeats into various categories is tedious, time-consuming, and could result in manual errors. An ML model is developed for accuracy, time efficiency with extensive data, and high learning.

### 2.3. Optimized, Subject-Wise Cross-Validated CNN Model

In this study, a prediction model is developed based on the SCA data from PhysioNet to identify and classify heartbeats as normal and abnormal and be applied to any new individual ECG recording.

**Figure 5:**
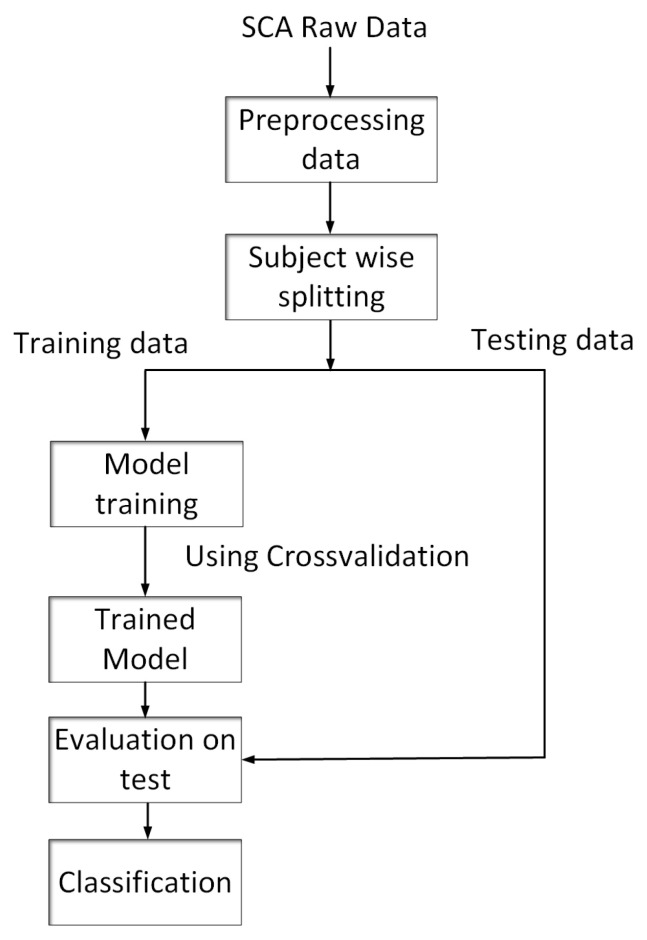
The process of building a CNN-based machine learning model.

Researchers have proposed different methods for identifying the risk of SCD using different markers in the literature. To predict SCA one hour before the VF onset, a kNN, SVM models were built [19,20,21]. Although these methods can be employed to predict SCA, the time required to extract features and the additional circuitry make them incompatible with real-time applications. The CNN model extracts features that are specific to the setting. Nguyen et al. built a CNN model only to extract features and, based on the output features, an SVM classifier was built to classify ECG segments. Five-fold cross-validation was performed to obtain an accuracy of 99.02% [22]. The accuracy is high because the same subject ECG data are present in the training and test sets. However, there is a low degree of sensitivity and specificity or negative predictive value. CNNs can learn non-linear features flexibly and have inherent feature-extraction capabilities. Moreover, CNN is effective at extracting spatial features from time series data. Hence, this work is focused on building an optimized, subject-wise cross-validated CNN model to classify SCA heartbeats. Building the CNN model involves the following steps: First, the SCA data are preprocessed. For each subject, Physionet’s ’ATR’ file contains ECG values, annotations, and frequency, and more information is sampled and scaled. We sampled the data at 500 ms and normalized the data. The next step is to split the dataset subject-wise. Then we optimize the model with subject-wise cross-validation. The next step is to deploy the model and evaluate its performance on test data. Implementing the model on a hardware platform is the final step. In Figure 5, the complete process of building the CNN model is represented. A half-hour recording of the patients in the SCA data shows a significant imbalance between the number of normal and abnormal heartbeats (the various abnormal beats listed in the table are grouped into one category and labeled as abnormal), with 96% beats as normal and only 4% as abnormal beats per subject. All abnormalities are combined to develop a binary CNN classifier. The weights are initialized based on abnormal beats rather than normal beats to address the class imbalance.

**Figure 6:**
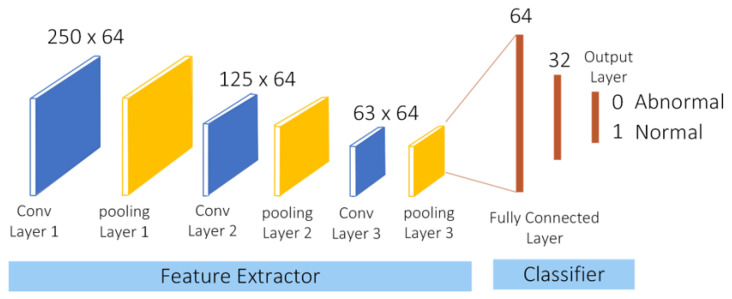
Layers of optimized CNN model.

Subject-wise cross-validation (CV) is a feature of a machine learning tool where the trained model learns from each piece of subject data, set by the learning rate parameter. For different databases, CNN architecture requires hyperparameter tuning. The hyperparameters, such as the number of layers, number of neurons, activation function, etc., offer a range of options. To obtain an optimized model, we tune the hyperparameters. The lower learning rate indicates slow but thorough training with a lower error rate. A slower-trained model is best suited for predictions on new subjects’ data that is raw and has not been previously trained. Hence, such a CV-based model is suitable for real-time applications [23]. This work was cross-validated subject-by-subject. The first step is to separate some subjects from the entire dataset for testing and evaluation. The remaining subjects’ data are divided into a training set and a validation set using the re-sampling method. We create a three-groups or three-fold (k = 3) dataset with different training and validation subjects. This is explained with an example; consider 12 subjects: 1, 2, 3, 4, 5, 6, 7, 8, 9, 10, 11, 12. The ECG samples of subjects two, five, and eleven were randomly selected for testing. The remaining subjects’ data were divided into three folds. In the first fold, 4 and 12 data are in the validation set, and 1, 3, 6, 7, 8, 9, and 10 are in the training set. On the second fold, 3 and 10 subjects are in validation, and 1, 4, 5, 6, 7, 8, and 9 of the 12 subjects are for training. On the third fold, there are 1 and 8 for validation and 3, 4, 5, 6, 7, 9, 10, 11, and 12 subjects in the training set. The fold (combination) that gives the model the highest performance is selected. Finally, an evaluation is performed to build an effective and efficient model on the test set. Training the ML model with subject-wise cross-validation in clinical settings is essential to simulate real-world medical diagnostics processes. This is because we train our model in such a way that the training set and test set (holdout set) have data from the same patients. At that time, the model will learn to classify the abnormal beats and pick up on identifying the characteristics of the patients. This will cause misjudgment in the classification error. Also, the data sample variability is significantly higher across patients than within patient data variability [24]. TensorFlow was used to implement the classification in a Python environment. As illustrated in Figure 6, our CNN includes three convolution layers and three pooling layers, and we added two fully connected layers and an output layer for classifying the heartbeats. The heartbeat is classified as either normal (1) or abnormal (0) heartbeat. As a precaution against overfitting, batch normalization and drop-out layers were added. The CNN model uses a rectified linear activation function (ReLU) to transform the summed weighted input from the node into the node’s activation or output for that input. A low learning rate model of 0.00001 is used for better training and validation. The batch size, which defines the number of samples propagated through the network, is set to a good default size of 32. A higher batch size (e.g., 64) reduces the error rate but increases the training time of the model. The values for these and other optimized CNN hyperparameter settings are listed in Table 3. Weights are initialized so that abnormal beats can be detected accurately. The effectiveness of the CNN model on SCA classification is evaluated using the following performance measures: precision, recall, confusion matrix, accuracy, and F1-score. The confusion matrix for the binary classification model is 2 × 2. The column of the matrix shows the true class, and the rows show the predicted class. The segment of the confusion matrix determines as *TP* (true positive) when the true class and predicted class values are normal (1), as *TN* (true negative) when the true class and predicted class values are abnormal (0). Thus, *TP*, and *TN* are diagonal elements. *FP* (false positive) is when the true value is normal (1), and the predicted value is abnormal (0), and *FN* (false negative) is the value for a model when the true value is abnormal and predicted value is normal. The diagonal cells show where the true class and the predicted class match. For a particular machine learning model, accuracy is a key performance parameter that indicates the rate at which anomalies are detected. Among the other performance, parameters are the true positive rate (Recall) and the true negative rate (Precision). F1 score, which is the geometric mean of recall and precision, is well-suited and used to compare the performance of ML models using different datasets. Following are the definitions and formulas: Classification accuracy is defined as the ratio of the number of correctly classified cases and is equal to the sum of *TP* (true positive) and *TN* (true negative) divided by the total number of cases *N*, where *N* = *TP* + *TN* + *FP* + *FN*, as given in the equation as Equation (1). The precision, recall and F1 score equations are given as Equations (2)–(4).

(1) $$\mathrm{Accuracy}=\frac{TP+TN}{TP+TN+FP+FN}$$

(2) $$\mathrm{Precision}=\frac{TP}{TP+FP}$$

(3) $$\mathrm{Recall}=\frac{TP}{TP+FN}$$

(4) $$F1\;\mathrm{score}=\frac{2}{Recall^{-1}+Precision^{-1}}$$

**Figure 7:**
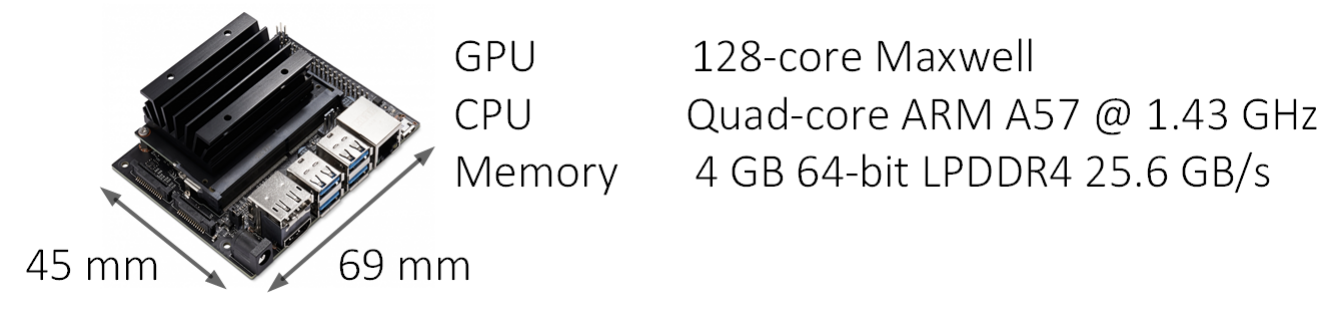
NVIDIA Jetson size and specification.

The receiver operating characteristic (ROC) curve also proves useful for evaluating machine learning techniques. A ROC curve is a graph showing the performance of a classification model at all classification thresholds. This curve plots two parameters, true positive rate, and false positive rate. The area under receiver operating characteristics (AUROC) tells how much the model can distinguish between classes. The higher the AUC, the better the model predicts 0 classes as 0 and 1 classes as 1. Thus, ML models built on different datasets can be compared based on the AUROC and F1 scores. The best-performing model is loaded on a platform with a high processing speed and portability. NVIDIA Jetson’s graphics processing unit (GPU) with quad-core ARM processor offers high performance and the ability to handle large datasets [25]. Therefore, the optimized CNN model with the best training accuracy is validated using the NVIDIA Jetson’s board. Figure 7 displays an NVIDIA Jetson Nano board, which has a portable size of 45 × 69mm^2^, a graphical processing unit (GPU), and memory of 4 GB.

## 3. Results and Discussion

### 3.1. Wireless Module

**Figure 8:**
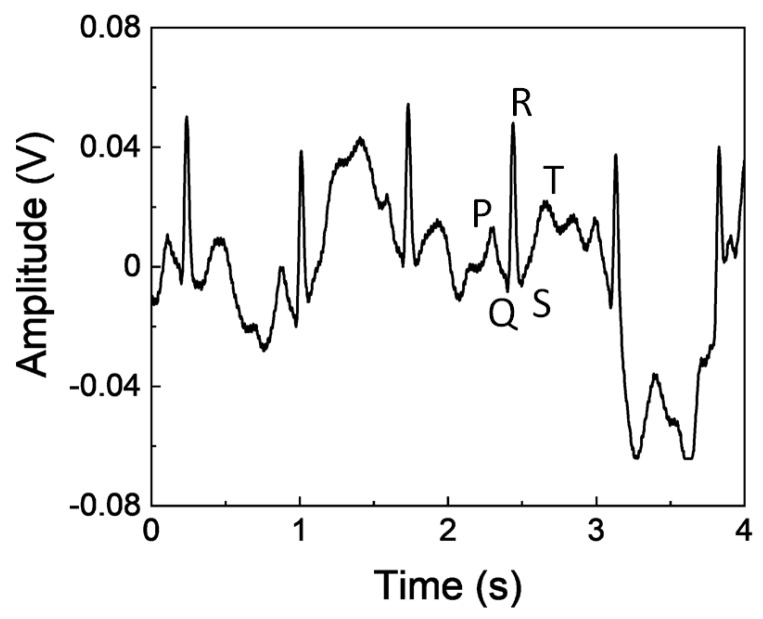
ECG signal acquired while the subject is walking.

An ECG signal from dry electrodes based on zeolites and PDMS is transferred to the proposed wearable wireless module using NI’s myDAQ. Before the participation of volunteers in this study, the necessary institutional review board (IRB) approvals were obtained. The raw ECG signal had a signal-to-noise ratio (SNR) of 7.21 dB. SNR is improved to 30.9 dB by filtering the power line noise frequency of 60 Hz and all higher-order frequencies above 100 Hz. The ECG signal obtained while the subject is walking is shown in Figure 8. It is observed that the peaks of *P*, *Q*, *R*, *S*, *T* are present. The denoising and baseline wandering are effectively taken care of using discrete wavelet transform MATLAB code [26].

**Figure 9:**
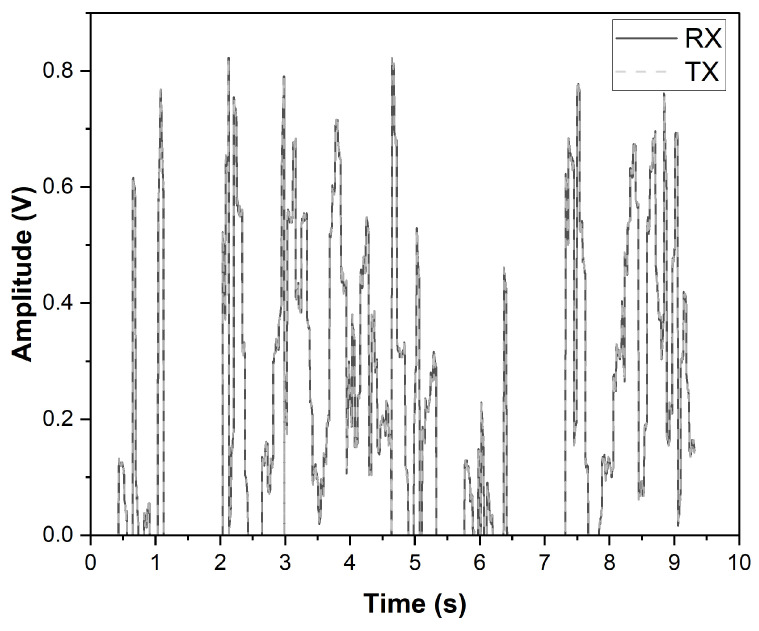
Comparison of the ECG signal at the transmitter and receiver.

The nRF24L01 transceiver wireless module transmits the ECG signal, while an EVAL board utilizes another nRF24L01 as a receiver. A distance of 5 m separates the EVAL board from the wireless transmitter. For the SCA database, the sampling frequency is 250 Hz, for which a data rate of 250 kbps is sufficient. A higher data rate can be used in the case of high sampling rate biosignals, but this will increase power consumption. While transmitting the digitized ECG signal at a rate of 250 kbps, the transceiver and the ADC module draws 3.4 mA of current for an ECG signal of 45 s, and the system with a 3.3 V supply has an average power consumption of 11.2 mW. The battery life can be estimated using the battery’s nominal capacity and the load’s average drawn current. The capacity of a battery is typically measured in milliamp-hours (mAh). For a 3.4 mA current (during transmission) and 240 mAh capacity battery (with CR032), the battery life of this wireless module is estimated to be 70 h. The SNR analysis is performed to determine the loss during transmission. For a 2m distance between the receiver and transmitter, the SNR of the ECG signal before transmission is 16 dB, and at the receiver is 6 dB, indicating a 10 dB loss. The plot of the ECG signal before and after the transmission is shown in Figure 9. Table 4 compares this work to similar works with the same supply voltage of 3.3 V. In comparison [27], our work has a 50% reduction in current and minimizes the power consumption. Using MATLAB code, the bit error rate is estimated between (0–0.1)%.

### 3.2. CNN Module

**Figure 10:**
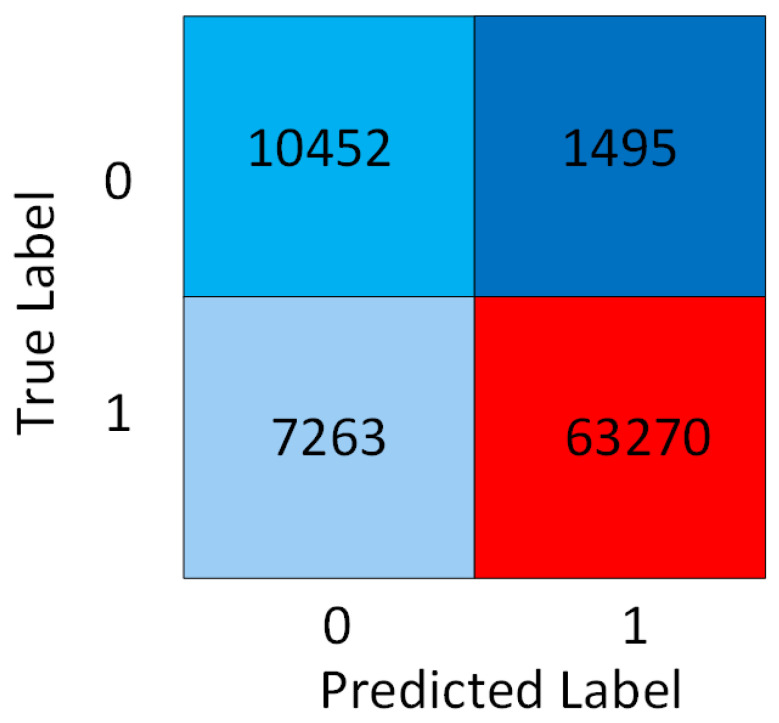
Confusion Matrix of the validation.

**Figure 11:**
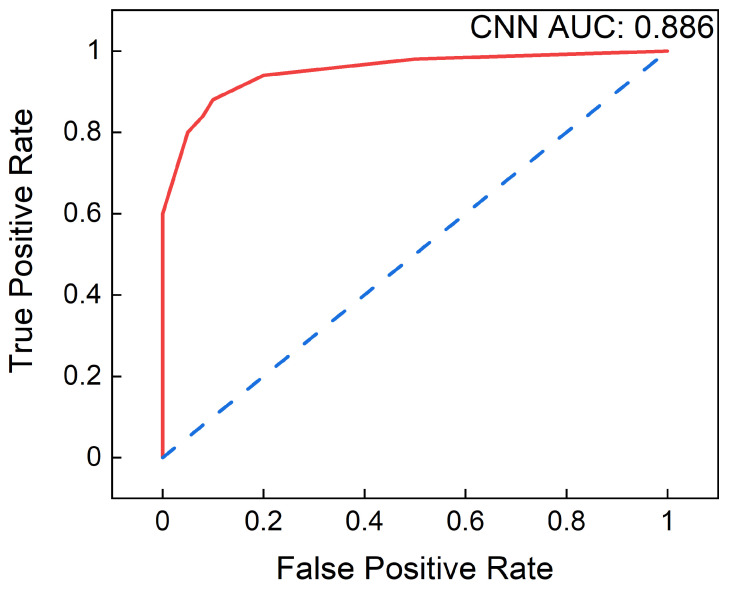
Area under the receiver operating characteristic curve of the CNN model.

The confusion matrix for the optimized CNN model is shown in Figure 10. Observation of the diagonal elements displays high numbers in the confusion matrix, indicating the good performance of the classifier model. The values of *TP* = 63,270, *TN* = 10,452, *FP* = 7263, and *FN* = 1495, respectively, based on the 2 × 2 confusion matrix. Based on the equations, the accuracy is calculated to be 89.3%. The CNN model implemented on NVIDIA Jetson has a similar degree of accuracy as the simulated model of approximately 89 percent, with a run time of 5.31 s. The precision, recall, and F1-score are calculated for normal and abnormal beats separately. Based on the equations, Table 5 lists the precision, recall, and F1 scores for the binary classification CNN model. The imbalanced dataset has a high F1 score of 0.94 for normal beat detection and 0.7 for abnormal beat detection. AUROC values for other machine learning work with different models and datasets are presented in Table 6. As can be seen from the AUROC value of our work, it is 0.7% higher than the prior proposed CNN models. An ML model’s receiver operating characteristic (ROC), the ratio of true positive rate to false positive rate, yields a plot as shown in Figure 11. The model performs well, as indicated by the ROC curve (AUC) area. We also have trained our CNN model with standard cross-validation techniques and tested it on unseen patient data. It gives a 0.755 AUC score, which is 0.131 lower than our proposed model. Thus, our CNN-based deep learning approach showed potential for better prediction of SCA beat annotations using a real-world database with imbalanced classes, avoiding the possibility of overfitting and implementing on the NVIDIA Jetson board.

## 4. Conclusions

A low-power wireless system is developed on ECG signal, acquired using zeolite-based electrodes, processed on an FR4 substrate-based read-out circuit. It is a compact system that can be used outside hospital settings to transmit ECG signals wirelessly. Using a wireless transceiver, the nRF24L01, which is compatible with any Arduino microcontroller, digitized ECG bits are transmitted and received over a distance of 5 m, with high signal integrity and a low bit error rate of ≤0.1%. With 250 kbps as the data rate, the transceiver consumes only 11.2 mW. If a CR032 battery is used, the design can run continuously for ≥70 h. To detect and identify abnormal beats from any new subject’s acquired ECG, a subject-wise cross-validated, three-fold CNN model is trained and implemented on an NVIDIA’s Jetson, yielding a prediction accuracy of 88%. Thus, an Arduino microcontroller and nRF24L01 transmitter are integrated with the analog front end (the zeolite-based ECG acquisition and COTS-based processing unit) incorporated with a CNN model on a portable NVIDIA Jetson is proposed in this paper.

## Figures and Tables

**Table 1:** Pin Configurations for nRF24L01 and Arduino.

Sl. No	nRF24L01 Pins	Arduino Pins
1.	GND	GND
2.	V${}_{CC}$	3.3 V
3.	C${}_{E}$	D7
4.	CSN	D8
5.	MOSI	D11
6.	MISO	D12
7.	SCK	D13
8.	IRQ	NC

**Table 2:** Annotations of heartbeats for SCA data.

Sl. No	Beat and Non-Beat Annotations	Description
1.	B	Bundle branch block beat
2.	E	Ventricular escape beat
3.	F	Fusion of ventricular and normal beat
4.	J	Nodal (Junctional) premature beat
5.	N	Normal beat
6.	S	Supraventricular escape beat
7.	V	Premature ventricular contraction
8.	|	Isolated QRS like artifact

**Table 3:** Optimized CNN hyperparameter setting.

Sl. No	Hyperparameters	Values
1.	Activation Function	ReLU
2.	Learning rate	0.00001
3.	Batch size	32
4.	Epochs	10–20
5.	Optimizer Algorithm	Adam

**Table 4:** Comparison with other nRF24L01 works at 3.3 V.

Reference	[28]	[27]	[11]	This Work
Current (mA) in TX	11.3	7.22	8.5	3.4
Current (mA) in RX	13.5	6.75	12.5	1.4
Data rate (Mbps)	2	2	(1–2)	0.25
Power (mW)	37.3	23.8	28	11.2
Distance (m)	NA	NA	NA	5
Bit Error Rate	NA	NA	NA	(0–0.1)%

**Table 5:** Testing results of the optimized CNN model.

	Precision	Recall	F1 Score
Abnormal	0.59	0.87	0.7
Normal	0.98	0.90	0.94

**Table 6:** Comparison with other ML works.

Reference	Dataset	Model	AUROC
[29,30]	EHR	Random Forest, LSTM	0.81, 0.88
[31]	UCI	CNN	0.86
This work	Physionet(SCA)	CNN	0.886

## Data Availability

Section Physionet SCA Data shares the information of the dataset in detail.
